# Effects of remaining dentin thickness on the bond strength of bleached dentin

**DOI:** 10.1186/s12903-020-01206-3

**Published:** 2020-08-05

**Authors:** Lei Jiang, Xiu-jiao Lin, Ying-hui Chen, Hao Yu

**Affiliations:** 1 Department of Prosthodontics, School and Hospital of Stomatology, Fujian Medical University, Fuzhou, China; 2 Research Center of Dental Esthetics and Biomechanics, Fujian Medical University, Fuzhou, China

**Keywords:** Dentin, Bleaching, Remaining dentin thickness, Shear bond strength

## Abstract

**Background:**

The bond strength of resin composites to dentin was reported to be related to either the remaining dentin thickness (RDT) or bleaching treatment. However, information is limited regarding the effects of RDT on the bond strength of bleached dentin. The present study aimed to investigate the effects of RDT on the microshear bond strength (μSBS) of resin cement to bleached dentin.

**Methods:**

A total of 120 dentin specimens were prepared and randomly divided into 2 groups: a bleaching group (group B) and a control group (group C). Hydrogen peroxide with a concentration of 35% (Ultradent, USA) was applied on the dentin surface for 2 × 1 d for group B, while no bleaching treatment was performed for group C. After the treatment, the specimens were finished and polished to obtain different RDTs (2, 1, and 0.5 mm) and divided into 3 groups of 20 specimens each. The bonding procedure was performed using Panavia V5 (Kuraray, Japan) with a bonding area of 0.785 mm^2^. For each group, half of the specimens were subjected to 5000 thermal cycles (subgroup T), while the other half did not receive thermocycling (subgroup N) (*n* = 10). The specimens were then subjected to the μSBS test using a universal testing machine. Data were analyzed by a three-way analysis of variance (α = 0.05). The fracture modes of the specimens were confirmed with a measuring microscope. Representative specimens with different fracture modes were observed with scanning electron microscopy (SEM).

**Results:**

The μSBS values were significantly affected by bleaching treatment (*p* < 0.001), whereas no significant effect was observed for thermocycling (*p* = 0.293). In terms of RDT, a significantly different μSBS value was found among the subgroups with different RDTs in group C (*p* = 0.003). However, the RDT did not significantly affect the μSBS values of bleached dentin in group B (*p* = 0.779). The μSBS values were significantly lower in group B than in group C (*p <* 0.001). A higher percentage of adhesive failure was observed in group B than in group C.

**Conclusion:**

Based on the present findings, it can be concluded that the RDT did not affect the bond strength of resin cement to bleached dentin.

**Clinical significance:**

Since RDT did not affect the bond strength of resin cement to bleached dentin, bonding procedures should not be performed immediately after intracoronal bleaching, even if the dentin is planned to be removed due to a tooth preparation process.

## Background

Tooth discoloration negatively affects the appearance, confidence, and quality of life of many people [1]. Various treatments, including tooth bleaching, tooth scaling and polishing, crowning and other restorations, have been recommended to restore the esthetics of discolored teeth [2, 3]. Among the abovementioned treatments, tooth bleaching has become increasingly popular because it is a safe, minimally invasive and cost-effective treatment [4–7].

Intracoronal bleaching, in which the bleaching agents directly contact the dentin, has been recommended for endodontically treated teeth with intrinsic discoloration [8]. Alternations in the surface morphology and structure of dentin [7, 9, 10], loss of mineral content [7, 9, 10], increase in dentin permeability [7], and metalloproteinase-mediated collagen degradation in dentin [7, 11] have been reported after intracoronal bleaching. Moreover, there is evidence that tooth bleaching may lead to a reduction in the bond strength of resin composites applied to previously bleached dentin [5, 12–16]. However, contrasting results regarding the bond strength of bleached dentin have been reported in the literature. Arcari et al. [17] investigated the microtensile bond strength of resin composites to bleached dentin and concluded that the bonding procedure for bleached dentin could be accomplished immediately after intracoronal bleaching. Similarly, the dentin bond strength remained unchanged after the treatments with 10% carbamide peroxide (CP) [18], 37% CP [8], and sodium perborate [19]. Given that the reduction in resin-dentin bond strength was thought to be mainly related to residual free radical breakdown from residual hydrogen peroxide [2, 6, 20, 21], different bleaching regimens might account for the discrepancy in the bond strength of bleached dentin in the abovementioned studies [18, 22].

In addition to the bleaching regimens, specimen preparation, such as the remaining dentin thickness (RDT), may play an important role in the bond strength of resin composites to bleached dentin [23–29]. It has been reported that the bond strength of resin composites to dentin is significantly correlated with the RDT. The thicker the RDT is, the higher the bond strength of the dentin [24, 25]. It could be postulated that the RDT influences the bleaching effects on the bond strength of resin composites to dentin and might be the cause of the inconsistencies in the previous reports. However, limited information is available in the literature.

Furthermore, aging protocols such as thermocycling, fatigue and water storage were reported to impair the bond strength of dentin [30–32]. Thermocycling, a widely used artificial aging method, may lead to contraction and expansion stresses at the adhesive interface and accelerate the chemical degradation of the adhesive interface, jeopardizing the bond strength [31]. Other studies revealed that thermocycling, conventional aging by means of water storage or accelerated aging by pH cycling resulted in a bond strength similar to the immediate bond strength [30, 33], inconsistent with the previously mentioned.

Therefore, the present study aimed to investigate the effects of different RDTs on the microshear bond strength (μSBS) between resin cement and dentin immediately after an intracoronal bleaching treatment. The following null hypotheses were tested: (1) that the RDT would not affect the bond strength of resin cement to bleached dentin, (2) that the bleaching treatment would not affect the bond strength of resin cement to dentin, and 3) that the bond strength of resin cement to bleached dentin would be the same before and after thermocycling.

## Methods

**Fig. 1:**
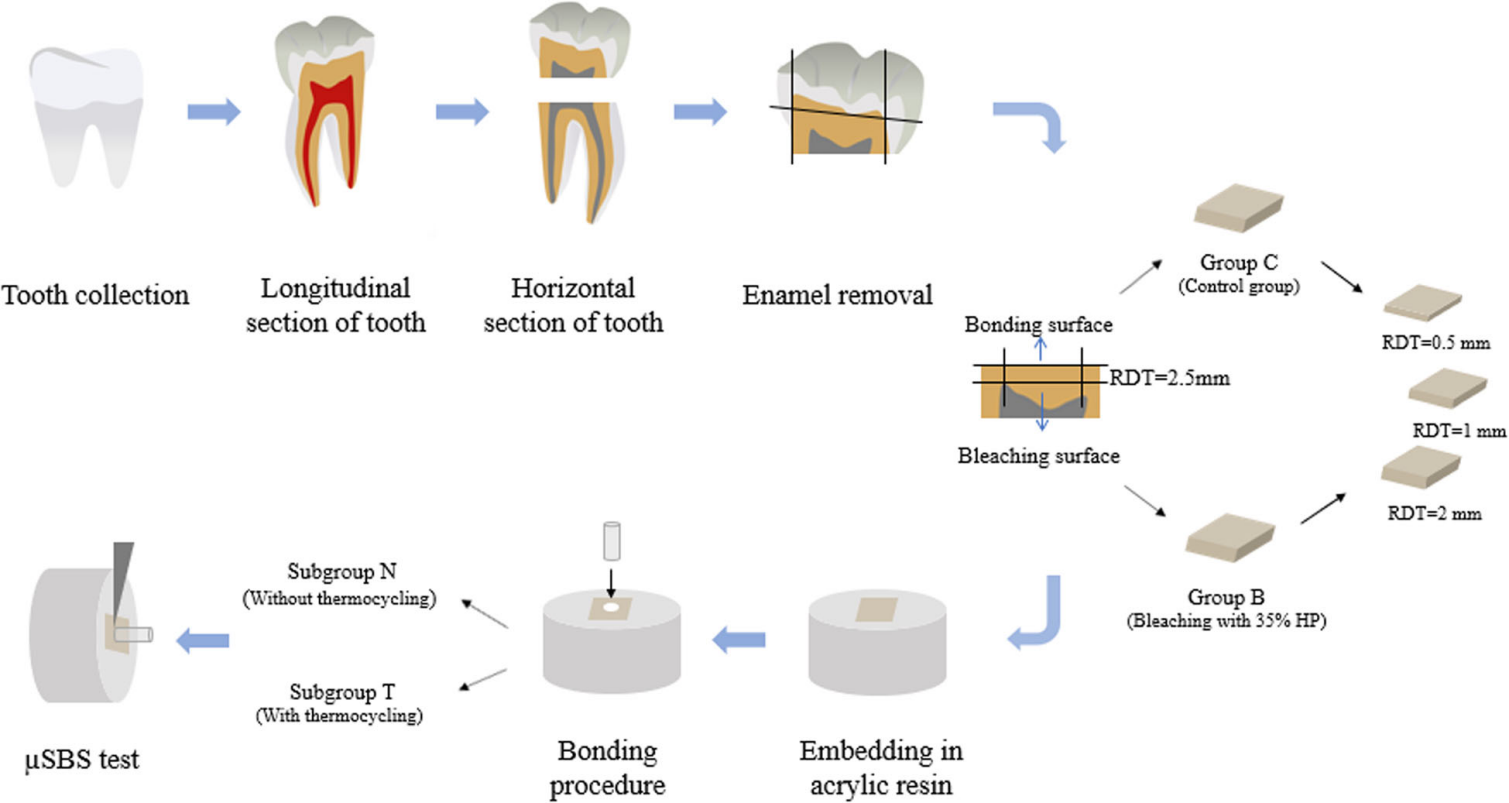
Experimental flowchart (RDT: remaining dentin thickness, HP: hydrogen peroxide)

The research protocol was reviewed and approved by the Research Ethics Committee at the School and Hospital of Stomatology, Fujian Medical University (No. 2015-CX-31). The experimental flowchart is shown in Fig. 1.

### Sample size calculation

Before the experiment, sample size was estimated by using G*Power software (Version 3.1.9.2. for Windows, G*Power, Dusseldorf, Germany). The effect size f, ɑ err prob., power, numerator df, and number of groups were set at 0.4, 0.05, 0.8, 2, and 3, respectively [34]. The results indicated that a minimal specimen number of 6 was required per subgroup.

### Specimen preparation

Dentin blocks were prepared from extracted noncarious human third molars from 18- to 35-year-old subjects of either gender. After extraction, the teeth were rinsed with saline solution immediately and immersed in 0.05% thymol solution at 4 °C before being tested. The teeth were divided into equal halves longitudinally and separated at the cementum-enamel junction (CEJ) using a low-speed diamond saw (Isomet, Buehler, Lake Bluff, USA) under running water. The coronal enamel was then removed from each half of the tooth at the dentinal-enamel junction (DEJ) to expose the dentin. The dentin specimens were obtained from the central region that was located between the pulp horns (center) of the coronal dentin [28]. The surfaces of the specimens were examined with a measuring microscope (MM400, Nikon, Tokyo, Japan) to ensure that no enamel existed. The dentin surface near the pulp chamber was called the bleaching surface and the dentin surface opposite to the bleaching surface was regarded as the bonding surface. The surface microhardness of the bonding surfaces of the dentin specimens was measured before bonding procedures, ensuring that the dentin blocks were prepared from similar regions. A microhardness tester with a Vickers diamond indenter was used to measure the baseline microhardness of all the specimens. Three indentations at intervals of 0.3 mm were made in the center of the moist specimen surface, with a force of 100 gf, lasting for 10 s. An average of the 3 values was then calculated and microhardness values within 63.47 to 67.25 kg/mm^2^ were selected [35].

One hundred and twenty dentin specimens were randomly divided into 2 groups: a bleaching group (group B) and a control group (group C). For group B, 35% hydrogen peroxide (Opalescence Endo, Ultradent Product Inc., South Jordan, Utah, USA) was applied to the bleaching surface for 2 sessions. For each session, bleaching gels were applied to the dentin surface at a thickness of 1 mm for 1 d. After the bleaching session, the bleaching gels were washed away from the specimen surfaces with distilled water. The dentin specimens were stored in an incubator at 37 °C (100% humidity) during the bleaching treatment. For group C, the specimens were stored in the incubator without the bleaching treatment. All the specimen surfaces were covered with wax except for the bleaching surfaces. Dentin specimens of groups B and C with the different RDTs (2, 1, and 0.5 mm) were then prepared by finishing and polishing at the bonding surfaces using 220-, 600-, 800-, and 1200-grit silicone carbide abrasive paper under continuous water cooling followed by ultrasonic irrigation for 2 min. Finally, 20 dentin specimens with each RDT were obtained (specimen dimensions: 3 × 3 × 2 mm, 3 × 3 × 1 mm, and 3 × 3 × 0.5 mm) for both groups B and C.

The dentin specimens were then embedded using acrylic resin (Ziran, Nissin, Hangzhou, Zhejiang, China). A silicone rubber cylinder mold with a diameter of 15 mm was used for the embedding process. Adhesive tape was used to protect the bonding surface from contamination by the acrylic resin. All specimens were stored in distilled water at 37 °C before the bonding procedures.

### Bonding procedures

The bonding was performed on the bonding surface of the specimens using the resin cement (Panavia V5, Kuraray dental, Niigata, Japan) according to the manufacturer’s instructions. After cleaning with pumice, Panavia V5 tooth primer (Kuraray dental, Niigata, Japan) was applied to the bonding surface and rubbed for 20 s. The primer was then dried and light cured for 10 s with a light-emitting diode (LED) light-curing unit (Elipar S10, 3 M ESPE, St Paul, MN, USA). A Teflon mold with an internal diameter of 1 mm and a height of 2 mm was placed on the bonding surface. The resin cement was then applied in the mold and light cured for 40 s with the LED light-curing unit. The excess materials were removed carefully with a sharp scalpel [2, 36]. The specimens were stored at room temperature for 1 h prior to the removal of the Teflon mold [2].

### Thermocycling

Each group was further divided into 2 subgroups (*n* = 10) according to the thermocycling procedure that was performed. In subgroup T, the specimens were subjected to 5000 thermal cycles that were split between water baths (dwell time 30 s) at 5 °C and 55 °C (TC-501F, Weier, Tianjin, China), while specimens in subgroup N did not receive thermocycling.

### μSBS test

Each bonded specimen was fixed on a universal testing machine (AGS-X, Shimadazu, Tokyo, Japan) and tested with a cross-head speed of 1 mm/min until failure occurred. The μSBS in megapascals (MPa) was calculated from the maximum load of failure in newtons (N) divided by the bonding area (0.785 mm^2^).

### Fracture mode analysis

After testing, the fracture surface was checked and confirmed under a measuring microscope at 40× magnification. The fracture modes were reported as follows: adhesive, failure at the adhesive-tooth interface in over 75% of the areas; cohesive, failure mainly in the resin cement such that over 75% of the tooth bonding area was covered with resin cement; and mixed, a combination of the abovementioned modes of fracture [12, 24, 37, 38]. The failure mode of each specimen was recorded as 1 of 3 types mentioned above. Representative images of the different failure modes were observed with scanning electron microscopy (SEM) (Quanta 250, FEI, USA).

### Statistical analysis

Statistical analyses were conducted by using SPSS software (version 20 for Windows, SPSS, Chicago, IL, USA). The assumption of normality was confirmed using the Kolmogorov-Smirnov test. Data were analyzed by a three-way analysis of variance (ANOVA) followed by Tukey’s multiple comparison test at α = 0.05.

## Results

The means and standard deviations of the μSBS values for different groups are shown in Table 1. The results of the three-way ANOVA revealed that the μSBS was significantly affected by bleaching (*p <* 0.001). For thermocycling, no significant difference was detected between the subgroups (*p* = 0.293). In terms of RDT, different effects were observed in group C and group B. A significant interaction was found between the bleaching treatment and RDT (*p* = 0.047). For group C, the μSBS values of the specimens with a 1 mm RDT were significantly greater than those with a 0.5 mm RDT (*p* = 0.004), while the μSBS values of specimens with a 2 mm RDT were similar to those with a 1 mm RDT (*p* = 0.858). However, for group B, no significant difference in the μSBS values was found among the groups with different RDTs (*p* = 0.779). Moreover, significantly lower μSBS values were observed in group B than in group C (*p <* 0.001).

**Table 1:** Means and standard deviations of the μSBS values (MPa) for different RDTs

RDT	Group	Subgroup	
		T (with thermocycling)	N (without thermocycling)
2 mm	Control	8.85 (2.60) ^A, a^	8.80 (3.92) ^A, d^
	Bleaching	4.92 (2.96) ^B, b^	3.04 (1.85) ^B, e^
1 mm	Control	7.68 (2.87) ^C, a^	11.41 (3.77) ^C, d^
	Bleaching	4.20 (1.99) ^D, b^	4.14 (2.74) ^D, e^
0.5 mm	Control	6.10 (2.51) ^E, c^	5.92 (3.86) ^E, f^
	Bleaching	2.73 (2.42) ^F, b^	4.57 (1.78) ^F, e^

Different uppercase letters in a row indicate significant differences in subgroups (*p* < 0.05). Different lowercase letters in a column indicate significant differences in different groups with different RDTs (*p* < 0.05)

*RDT* Remaining dentin thickness

**Fig. 2:**
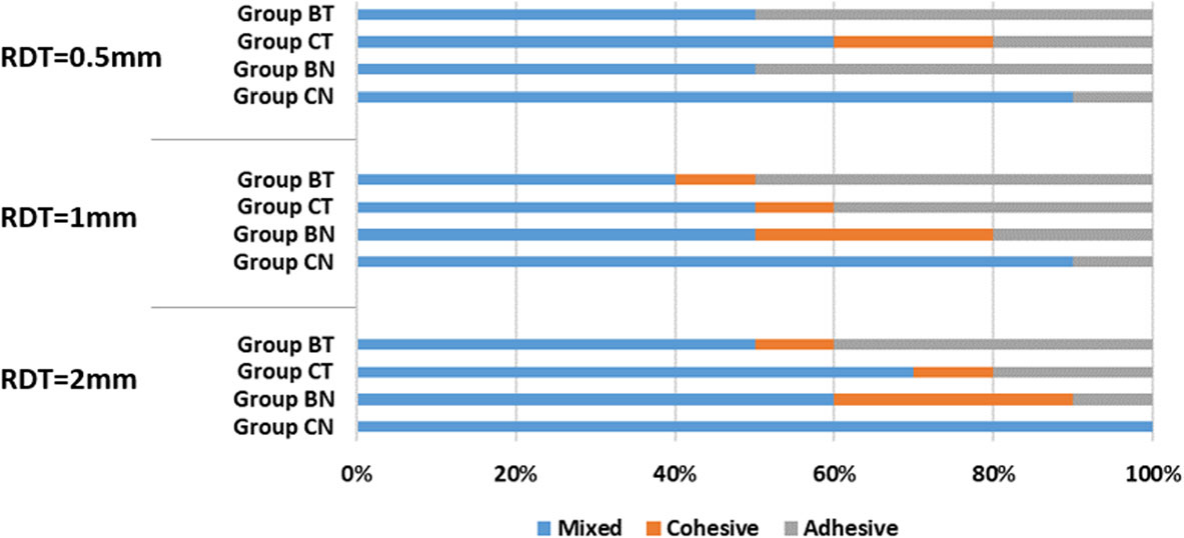
Distribution of failure modes among different RDT groups (RDT: remaining dentin thickness)

**Fig. 3:**
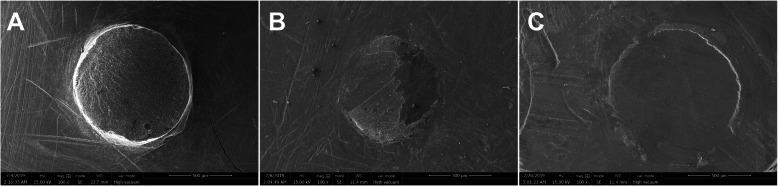
Representative SEM images of specimens with different failure modes: (**a**) Mixed failure; (**b**) Cohesive failure; (**c**) Adhesive failure

For all specimens, 26.7% showed adhesive failure, 10.0% showed cohesive failure in the resin cement, and 63.3% showed mixed failure. Figure 2 illustrates the frequencies of the different failure modes in each group. Representative images of the adhesive interfaces are shown in Fig. 3. A predominance of the mixed failure model was found in group CN (control group without thermocycling), followed by adhesive failure. An increased percentage of adhesive failure was obtained in group BN (bleaching group without thermocycling) and group CT (control group with thermocycling). A similar trend in the fracture mode was found among the specimens with different RDTs.

## Discussion

Based on the present findings, the null hypothesis that the bleaching treatment would not affect the bond strength of the resin cement to the dentin was rejected. The results failed to reject the null hypotheses that the RDT would not affect the bond strength of resin cement to bleached dentin and that the bond strength of resin cement to bleached dentin would be the same before and after thermocycling.

In previous studies, the effect of RDTs, ranging from 0.5 to 4.5 mm, on bond strength was investigated [24, 25]. However, it has been reported that 64–76% of the tooth structure is removed after the tooth preparation process [39]. An RDT of 2 mm is considered ideal, while an RDT lower than 2 mm is common in clinical situations [39]. Therefore, RDTs of 2 mm, 1 mm and 0.5 mm were selected in the present study.

The bond strength detected in the present study ranged from 5.92 to 11.41 MPa in the control group and 2.73 to 4.92 MPa in the bleaching group, respectively, which was within the range reported previously [13, 31, 37, 40]. The minor difference may be due to the different origins of the dentin specimens and adhesive systems used [33, 41, 42]. A significant reduction in the bond strength of bleached dentin was observed when compared with that of unbleached dentin, which is in accordance with previous studies [6, 8, 9, 12, 13, 37]. The residual oxygen due to the breakdown of hydrogen peroxide was deemed to be the most relevant cause [12]. Bleaching agents could significantly increase metalloproteinase-mediated collagen degradation in dentin even after only 24 h [7], jeopardizing the bond strength [43]. Morphological and compositional changes (e.g., loss of calcium and alterations in the organic substance porosity) in the bleached dentin may weaken the adhesive interface and compromise the bond strength [9, 10]. Moreover, the dehydration of the dentin due to the application of bleaching agents [44] might cause the collagenous fiber network to collapse [45], thus compromising the bond strength of the bleached dentin [28, 46].

The present study detected a significantly decreased bond strength in the deep dentin without the bleaching treatment, which is consistent with previous reports [23–26, 29, 47]. Deep dentin has much less intertubular dentin than superficial dentin, while intertubular dentin plays a key role in the resin-dentin bonding [25, 26]. In addition, a recent review demonstrated that RDT significantly affects the bond strength of dentin, which may be attributed to regional differences in wetness [42]. Interestingly, no significant difference in the bond strength was found among the specimens with different RDTs after bleaching. The low molecular mass of H_2_O_2_ (34 Da) favors its rapid and powerful diffusion into detin tubules, even enamel prisms, and may have retained hydrogen peroxide or oxygen radicals for an undetermined length of time [48]. In combination with the fact that intracoronal bleaching not only reduced the microhardness of the dentin but also reduced that of the enamel [49], it may be assumed that a relatively even amount of residual oxide remained at different levels of the dentin after the intracoronal bleaching treatment, exerting a more powerful effect than that of RDT on dentin bond strength. Residual oxygen could either interfere with resin infiltration into primed dentin or inhibit the polymerization of resin composites [12], resulting in a decreased bond strength.

Most previous studies measured the bond strength of bleached dentin without thermocycling [8, 9, 13, 18, 24–26, 28, 29]. Thermocycling is the in vitro process of subjecting a restoration and tooth to temperature limits similar to those experienced in the oral cavity [30]. It would be important to evaluate the influence of thermocycling on the shear bond strength of the bleached dentin. The cycles of thermocycling in the present study were determined according to previous studies [2, 30] and may be considered as a simulation of 6 months in vivo [50]. In the present study, the bond strength of coronal dentin with different RDTs remained unchanged after thermocycling regardless of whether they were bleached. A similar result was reported by Korkmaz et al. [30]. It is important to note that the 5000 thermal cycles may be insufficient to cause changes in the μSBS values [30]. A further study with increased cycles to explore the influence of thermocycling on the bond strength of dentin should be performed. With regard to the failure mode, an increased percentage of adhesive failure was exhibited in the bleaching group, which is in agreement with previous studies [12, 37].

The present study has several limitations. First, the μSBS test should be performed with several 1-mm resin bonds on the dentin, and an average should describe the real value for the tooth. Second, the dentin tubules of molars were in different directions regarding the pulp chamber and may affect the results [42].

Based on the present results, the bond strength of bleached dentin was compromised regardless of the RDT. According to previous studies, a waiting time of 1–3 weeks before a bonding procedure [2, 4], the application of an antioxidant (e.g., sodium ascorbate) [6, 51–53], and laser irradiation [37, 53] may be recommended to restore the compromised bond strength of the bleached dentin. However, further in vitro and in vivo studies are needed to confirm these hypotheses.

## Conclusions

The RDT and thermocycling did not affect the bond strength of the resin cement to the bleached dentin, while the bond strength of the resin cement to the dentin was negatively affected by the bleaching treatment. Therefore, bonding procedures should not be performed immediately after intracoronal bleaching, even if the dentin is planned to be removed due to a tooth preparation process.

## Data Availability

Further data may be requested by contacting the corresponding author. We declare that any data regarding the study will easily be provided.

## Abbreviations

μSBS: Micro-shear bond strength
RDT: Remaining dentin thickness
ANOVA: Analysis of variances
